# Preparation of Silk Fibroin–Carboxymethyl Cellulose Composite Binder and Its Application in Silicon-Based Anode for Lithium-Ion Batteries

**DOI:** 10.3390/nano15191509

**Published:** 2025-10-02

**Authors:** Shuai Huang, Ruyi Wang, Mingke Lei, Qingxuan Geng, Qingwei Li, Jiwei Zhang, Jingwei Zhang

**Affiliations:** 1 School of Environmental Engineering, Yellow River Conservancy Technical University, Kaifeng 475004, China; 2 Henan Engineering Technology Research Center of Green Coating Materials, Kaifeng 475004, China; 3 National & Local Joint Engineering Research Center for Applied Technology of Hybrid Nanomaterials, Henan University, Kaifeng 475004, China; wrysqq@163.com (R.W.);; 4 State Key Laboratory of Green Papermaking and Resource Recycling, Advanced Research Institute for Multidisciplinary Science, Qilu University of Technology (Shandong Academy of Sciences), Daxue Road 3501, Jinan 250307, China

**Keywords:** Si, lithium-ion battery, binder, carboxymethyl cellulose, silk fibroin

## Abstract

The molecular structure and mechanical resilience of the binder are crucial for mitigating volume expansion, maintaining electrode structural integrity, and enhancing the cycling stability of silicon-based anode materials in lithium-ion batteries. In this study, from the perspective of binder molecular structural design, commercial carboxymethyl cellulose (CMC) was modified with silk protein (SF), which has good mechanical properties and abundant surface functional groups, to address issues such as high brittleness, poor compliance and easy cracking of the electrode structure during charge and discharge cycles, and to enhance the mechanical properties of the CMC-based binder and its interaction with silicon particles, so as to improve the cycle stability of silicon-based materials. The mechanical properties of the CMC binder were significantly improved and the interaction between the binder and the surface of the silicon particles was enhanced by the addition of SF. When the SF content was optimized at 6 wt%, the electrode exhibited the best electrochemical performance, delivering a specific capacity of 1182 mAh/g at a high current density of 5000 mA/g, and retaining a capacity of 1138 mAh/g after 50 cycles at 1000 mA/g, demonstrating excellent electrochemical durability.

## 1. Introduction

Silicon (Si), with high theoretical specific capacity (4200 mAh/g), low reaction potential, and abundant natural resources, is one of the preferred anode materials for the next generation of high-energy-density lithium-ion batteries [1,2,3]. However, there is a significant volume change (>300%) in the process of lithiation and delithiation of Si anodes, which leads to particle breakage, pulverization, electrode structure destruction, unstable solid electrolyte interface (SEI) and other problems under stress [4,5,6,7], limiting its further commercial application.

Electrodes can be regarded as a kind of composite material, primarily composed of active materials, conductive agents, and binders. Although the binder constitutes only a small fraction of the electrode composite, it plays a crucial role in the electrochemical performance of electrode materials with a volume effect [8,9,10,11,12]. Through rational molecular design, the binder can enhance binding between the silicon particles, conductive carbon black, and the current collector, thereby facilitating the formation of an effective conductive network throughout the electrode. A binder solution with good dispersion can evenly and effectively disperse the active substances and conductive carbon black inside itself, avoiding clusters and sedimentation. Secondly, the adhesive can bond the active substance, conductive carbon black, and collector fluid together to form a stable structure, so that they can form good electrical contact with each other. Finally, during repeated charging and discharging, the binder acts as a buffer to alleviate volume expansion and structural damage of the active material and maintain the stability of the electrode and the integrity of the electronic channel, thereby improving cycling and rate performance.

Carboxymethyl cellulose (CMC), as a commonly used water-based binder for silicon-based materials, has the advantages of good stability, low price, safety, environmental protection, strong interaction with silicon, good dispersion of conductive agent, etc. [13,14,15], but CMC is weak in viscosity, high in brittleness, poor in flexibility, and easy to crack when charging and discharging [16,17]. In order to better adapt to the volume effect of Si-based anode materials, further modification research is needed [18,19,20,21,22]. Silk fibroin (SF) has a long molecular chain and contains rich functional groups such as amino, hydroxyl, and carboxyl. These functional groups can interact with CMC, and SF has good mechanical properties, flexibility, and tensile strength. Meanwhile, the β-sheet crystalline domains in SF can act as physical crosslinking points, significantly enhancing the binder’s rigidity. A composite binder composed of CMC-SF can alleviate the volume expansion and contraction of the Si-based anode materials during the charge–discharge process, thus improving its cycle stability. Zhang et al. [23] reported a biopolymer composite binder composed of rigid poly (acrylic acid) (PAA) and flexible SF tailored for micro-sized silicon anodes. The PAA/SF binder shows robust gradient binding energy via chemical interactions between carboxyl and amide groups, which can effectively accommodate a large volume change in the silicon. This PAA/SF binder also shows much stronger adhesion force and improved binding with high-surface/defective carbon additives. The Si/C electrode with a PAA/SF binder delivers a specific capacity of approximately 2000 mAh/g under 0.2 C, after which the rate performance decreases rapidly, decaying to mAh/g at 1 C. The specific capacity is mAh/g with an average CE of 99.6% after 500 cycles at 0.5 C, which is higher than those of PAA-based electrodes. Choi et al. [24] propose a new binding mechanism, i.e., a conformational change induced by SF to address these issues. Triggered by PAA, SF undergoes a conformational change from an amorphous phase to a β-sheet-rich structure and constructs a hierarchically ordered framework of PAA-SF, which provides excellent mechanical strength through the formation of densely packed structures. Additionally, the unique β-sheet-rich structure of the PAA-SF binder could facilitate lithium-ion diffusion via coordination bonds with lithium ions. Consequently, the PAA-SF binder effectively mitigates stress from the volume expansion, enhances the rate capability, and constructs a stable SEI layer, thereby extending the cycling stability of silicon anodes. The cells within this binder can sustain a high reversible specific capacity of approximately 2500 mAh/g at a current density of 420 mA/g, approximately 1200 mAh/g at a current density of 4200 mA/g. After 200 cycles at 2100 mA/g, its specific capacity was 1500 mAh/g. Currently, there have been no reports on the use of CMC and SF (silk fibroin) composite binders to mitigate the volume effect of silicon-based materials. Therefore, incorporating SF into the CMC binder system is expected to significantly improve its flexibility and elasticity, enhance interfacial adhesion with silicon particles, and effectively suppress the wetting behavior of CMC in organic electrolytes.

In this study, we designed and fabricated a novel SF-modified CMC binder to alleviate the volume expansion of silicon during charge/discharge cycles. This modification strategy synergistically enhanced the interfacial adhesion between CMC matrices, silicon nanoparticles, and current collectors through the following mechanisms: (1) the abundance of polar functional groups (-NH_2_ and -OH) in SF facilitated strong hydrogen bonding and chemisorption with both CMC chains and silicon surfaces; (2) the viscoelastic SF network effectively accommodated the large volume change of the silicon anodes during lithiation/delithiation cycles; (3) the intertwined SF-CMC composite structure significantly improved the mechanical integrity and flexibility. Consequently, the optimized binder system exhibited markedly enhanced electrochemical performance. This design paradigm presents a viable strategy for developing next-generation polymer binders capable of accommodating high-strain electrode materials, providing critical insights for addressing persistent challenges in high-energy-density battery systems.

## 2. Materials and Methods

### 2.1. Preparation of Silk Fibroin (SF) [25]

Before preparing the SF, the degummed silk fibers were first prepared. Following the typical process, raw Bombyx mori silk cocoons (2 g) were first cut into fragments and subjected to alkaline degumming in 5 wt% anhydrous Na_2_CO_3_ aqueous solution (bath ratio 1:50, *w*/*w*). Then, this system was refluxed at 100 °C for 30 min under mechanical agitation, followed by thorough rinsing with deionized water to remove the residue of the Na_2_CO_3_ solution; the resulting degummed silk fibers were vacuum-dried at 60 °C for 12 h.

To prepare the SF, the above-mentioned degummed silk fibers (2 g) were dissolved in 9 mol/L lithium bromide (LiBr) aqueous solution under continuous magnetic stirring to achieve a homogeneous solution. Then this solution was transferred into a cellulose dialysis bag (MWCO: 13 kDa) and dialyzed against ultrapure water. Dialysis completion was verified by the absence of Br^−^ ions (0.1 mol/L AgNO_3_ assay). After dialysis was completed, the SF solution was centrifuged (6000 rpm, 5 min) to remove insoluble residues. Then, after freeze-drying this solution, the SF protein was obtained.

### 2.2. Preparation of CMC-SF Composite Binders

**Figure 1:**
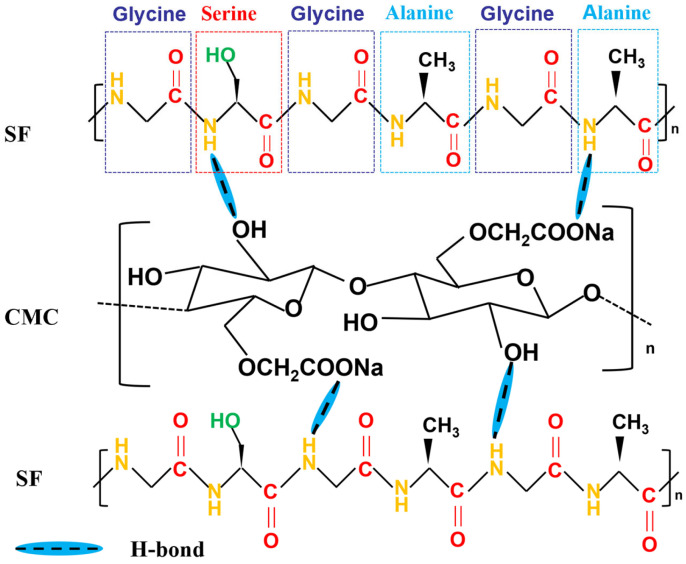
Schematic illustration of chemical interactions of CMC and SF binder.

A 2 wt% CMC aqueous solution was prepared under magnetic stirring. SF powder was dissolved in formic acid to form a 2 wt% SF solution. Three CMC-SF formulations were synthesized by blending SF solution into CMC matrix at mass ratios of 3%, 6%, and 9% (SF/CMC, *w*/*w*), designated as CMC-SF-3, CMC-SF-6, and CMC-SF-9, respectively. The mixtures were homogenized using a planetary centrifugal mixer for defoaming and thorough blending to ensure molecular-level integration and eliminate air bubbles. The SF structure, dominated by glycine (∼45%), alanine (∼30%), and serine (∼12%), facilitates hydrogen bonding between CMC’s –COO^−^ groups and SF’s –NH– moieties (–COO^−^···^+^H3N–), resulting in a flexible, self-crosslinked polymeric network. Figure 1 illustrates the intermolecular interactions between CMC and SF. The overall flowchart is shown in Figure S1.

### 2.3. Structure Characterization of CMC-SF Composite Binders

Fourier-transform infrared spectroscopy (FTIR) was performed using a VERTEX 70 spectrometer (Bruker Optics, Ettlingen, Germany) with a wavelength range of 4000–400 cm^−1^ with 4 cm^−1^ resolution. X-ray powder diffraction (XRD) analysis was conducted using a DX-2700BH diffractometer (Dandong Haoyuan Instrument Co., Ltd., Dandong, China) to evaluate the crystallinity of the samples. The samples were prepared as freeze-dried solid powders, and the measurements were carried out at a voltage of 40 kV and a current of 30 mA. Rheological properties and viscosity measurements were performed using a DHR2 rotational rheometer (Waters Corporation, Milford, MA, USA). Peel strength tests were evaluated using an Instron 5965 universal tester (Norwood, MA, USA).

### 2.4. Electrochemical Performance Characterization

The active material used in the experiment was 80 nm silicon powder, and the conductive agent was carbon black (Super-P). The silicon powder and conductive carbon black were thoroughly ground and mixed in an agate mortar. An appropriate amount of binder (mass ratio of silicon powder, carbon black, and binder = 5:3:2) was added, and the mixture was transferred to a defoaming box. Distilled water was added as a solvent, and the mixture was homogenized using a defoaming machine. The resulting slurry was uniformly coated onto a copper foil using a doctor blade with a gap of 100 μm. The coated samples were dried in a vacuum oven at 80 °C for 12 h. Finally, they were cut into round slices to prepare the electrodes; the active material mass loading (areal density) for each electrode was as follows: CMC binder (1.27 mg/cm^2^), CMC-SF-3 binder (1.25 mg/cm^2^), CMC-SF-6 binder (1.32 mg/cm^2^), and CMC-SF-9 binder (1.35 mg/cm^2^). Battery assembly was performed in an argon-filled glove box (H_2_O and O_2_ content ≤ 1 ppm) using 2032-coin cell components. Lithium metal was used as the counter electrode and the prepared electrode as the working electrode, and the electrolyte consisted of a 1:1:1 volume ratio of EC: DEC: DMC with 1 mol/L LiPF_6_ as the solute. A microporous polypropylene membrane (Celgard 2400, Charlotte, NC, USA) was used as the separator.

Galvanostatic charge–discharge tests were conducted using a battery testing system (CT-2001, Wuhan Lanhe Electronics Co., Ltd., Wuhan, China) within a voltage range of 0.01–1.5 V. Cyclic voltammetry (CV) measurements were performed using a CHI electrochemical workstation (Chenhua Instruments, Shanghai, China) with a scanning voltage range of 0.01–1.5 V and a scan rate of 0.1 mV s^−1^. Electrochemical impedance spectroscopy (EIS) was carried out using an XM Energylab electrochemical workstation (Solartron Analytical, Farnborough, UK) under the following conditions: a frequency range of 0.1 Hz to 100 kHz and an amplitude of 5 mV.

## 3. Results and Discussion

### 3.1. Subsection Chemical Interactions Between CMC and SF

**Figure 2:**
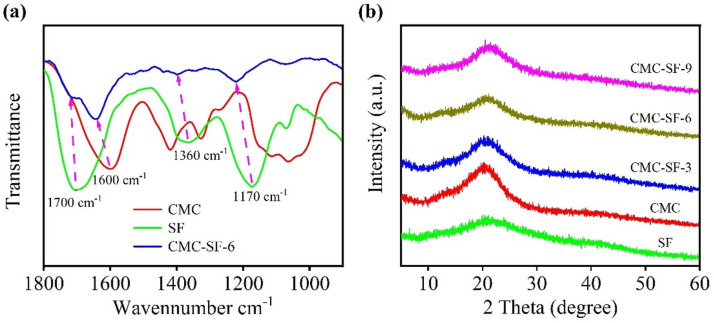
Infrared spectrum of SF, CM, and CMC−SF-6 (**a**); XRD patterns of CMC−SF binders with different ratios (**b**).

SF contains a large amount of amino acids, mainly glycine (45%), alanine (30%), and serine (12%). These amino acids are rich in amino and hydroxyl groups, which can form hydrogen bonds with CMC and nano-silicon particles during the blending process. Figure 2a shows the FTIR spectra of CMC, SF, and the prepared CMC-SF samples. The main absorption peaks of the prepared SF solution appear at 1700 cm^−1^, 1360 cm^−1^, and 1170 cm^−1^, corresponding to the C=O stretching vibration of amide I, the N-H bending vibration of amide II, and the C-N stretching vibration of amide III, respectively [26]. For CMC, the characteristic peak at 1600 cm^−1^ corresponds to the C=O stretching vibration, while the peaks at 1417 cm^−1^ and 1330 cm^−1^ are attributed to C-H vibrations, and the peak at 1070 cm^−1^ corresponds to the C-O-H stretching vibration [27]. In the CMC-SF composite binder, the amide-related peaks are clearly present, and their positions are shifted to higher wavenumbers, specifically to 1713 cm^−1^, 1390 cm^−1^, and 1219 cm^−1^, respectively. In comparison, the C=O stretching vibration of the CMC composite binder shifts to 1657 cm^−1^. These peak shifts in the characteristic peaks in the FTIR spectra of the CMC-SF composite binder indicate interactions between SF and CMC, leading to the observed peak shifts. Figure 2b shows the XRD patterns of CMC-SF with blending ratios. Both the SF and the CMC-SF binders show a broad diffraction peak near 20°, indicating an amorphous structure. Compared with the diffraction pattern of pure CMC, the addition of SF does not change the amorphous nature of the binders.

**Figure 3:**
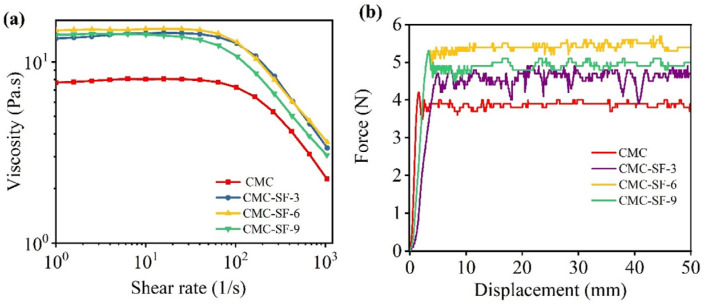
Variation in viscosity with shear rate of CMC-SF binders with different ratios (**a**); Force–displacement curve (**b**).

Figure 3a illustrates the apparent viscosity of sample solutions as a function of the shear rate. As the SF content increases, the viscosity of the CMC-SF samples rises rapidly, reaching its maximum when the SF content is 6%. When the SF content increases to 9%, the viscosity becomes comparable to that of the 3% SF sample. This indicates that the interaction between SF and CMC leads to a significant increase in viscosity. At shear rates below 10^2^ s^−1^, all solutions exhibit a constant viscosity regardless of the shear rate. At higher shear rates, a pronounced shear-thinning effect is observed. However, the viscosity of samples containing SF remains higher than that of pure CMC. The enhanced viscosity can help stabilize the electrode slurry.

The adhesion and mechanical properties of binder materials are critical physical characteristics. The peel strength of electrode sheets reflects the bonding strength between the binder and the active material as well as the current collector. To evaluate this, peel strength tests were conducted. Figure S2 shows a comparison of samples before, during, and after the peel test. From the comparison, it can be observed that the CMC sample was almost completely detached from the current collector after peeling, with significant exposure of the copper foil. This indicates that the adhesion between the CMC and the Si nanoparticles as well as the copper foil was poor, failing to effectively bind the active material to the copper foil. In contrast, the CMC-SF samples modified with SF showed significant improvement in adhesion after the peel test. For the CMC-SF samples, only minor detachment occurred, with localized exposure of the copper foil. From the images during the testing process, the amount of active material adhering to the double-sided tape initially decreased and then slightly increased with the addition of SF, suggesting a trend in the adhesion performance. From the force–displacement curves in Figure 3b, it can be observed that the CMC-SF samples modified with SF exhibited higher peel strength compared to pure CMC. The test results showed that the peel force for CMC was approximately 3.9 N, which was the lowest among the tested samples. The peel forces for CMC-SF-3, CMC-SF-6, and CMC-SF-9 samples were 4.5 N, 5.2 N, and 4.9 N, respectively. The peel strengths of all SF-modified CMC samples were increased, with the CMC-SF sample containing 6% SF showing the highest peel strength. When the SF content was further increased, the peel strength slightly decreased, which was likely due to the excessive aggregation or formation of aggregates of SF and their reduced compatibility with CMC. At an appropriate SF content, SF can be uniformly dispersed within the CMC matrix and form a dense “bridge network” between the CMC molecular chains and the silica particle surface through hydrogen bonding. However, as the SF content increases, SF molecules may undergo excessive aggregation or form larger aggregates. These aggregates can affect the uniformity of the binder, leading to a reduction in adhesion to the electrode surface and subsequently decreasing the peel strength.

Figure S3 shows isothermal dynamic frequency sweep tests on the composite binder. The SF-modified CMC binder exhibits a higher storage modulus than the loss modulus (G′ > G″) across the entire frequency range, indicating that the composite binder possesses a three-dimensional crosslinked network structure and demonstrates predominantly elastic behavior at all frequencies.

Based on the above characterization results, it is evident that the CMC-SF binder modified with SF, compared to unmodified CMC, benefits from the abundant amino and hydroxyl functional groups in SF. These groups interact with the carboxyl groups on the CMC chains, and there are also strong interactions between the hydroxyl groups on the Si surface and the free carboxyl and amino groups. These interactions contribute to the enhanced adhesion and mechanical properties of the CMC-SF binder.

The stability of the binder in the electrolyte is a crucial factor in determining the structural stability of the electrode in the electrolyte. The stability of the binder in the electrolyte can be tested by measuring its mass change after immersion and solubility. If the mass change in the binder increases, it means that more electrolyte was absorbed, and excessive electrolyte will damage the structure of the binder, leading to separation between the active material and the current collector, thus compromising the integrity of the electrode structure and causing electrode instability. On the other hand, if the binder has low mass change upon electrolyte absorption, it means that the electrolyte absorption was limited, which prevents the electrode from being properly wetted by the electrolyte. This results in slower lithium-ion transport and an increased internal resistance of the battery.

To evaluate the solubility qualitatively, the binder can be soaked in the electrolyte for a certain period, and the state of the binder can be observed and compared. The method to test the binder’s mass change upon electrolyte absorption involves preparing the binder into a thin film. After weighing, the film is placed in the electrolyte at a constant temperature for a certain period, then removed. The electrolyte on the surface of the film is wiped off, and the film is weighed again. The percentage difference in weight between the two measurements can be used to represent the electrolyte absorption of the binder. The calculation formula is as follows:

Percent Mass Change = (W_1_ − W_0_)/W_0_ × 100%

where W_0_ is the weight of the film before immersion in the electrolyte; W_1_ is the weight of the film after electrolyte absorption (g).

The percent mass change in different binders upon electrolyte absorption is shown in Table 1. The test results indicate that the percent mass change in CMC is relatively high at 8.12%. As SF is added, the percent mass change in CMC decreases. When 3% SF is added, the percent mass change is 6.93%, and when 6% SF is added, the percent mass change drops to 5.86%. When 9% SF is added, the percent mass change further decreases to 5.13%. The percent mass change generally decreases as the amount of SF increases. A higher percent mass change causes structural damage to the electrode during cycling, which can affect the electrochemical performance. After modification, the percent mass change in the CMC-SF binder decreases, which helps to enhance the structural stability of the electrode. Furthermore, contact angle measurements demonstrate that a higher SF content reduces the contact angle, indicating improved electrolyte wettability (Figure S4) This is consistent with the percent mass change results of the binder in the electrolyte.

### 3.2. Electrochemical Performance

To evaluate the electrochemical stability of the prepared binder within the charge/discharge voltage range of the electrode, the binder was fabricated into electrode sheets without adding active materials or conductive additives, and coin cells were assembled using lithium metal as the counter electrode. Subsequently, CV tests were conducted to characterize whether the prepared binder participated in electrode reactions. As shown in Figure S4, compared with CMC, the prepared sample exhibited no oxidation or reduction peaks throughout the entire charge/discharge voltage range, indicating its favorable electrochemical stability.

**Figure 4:**
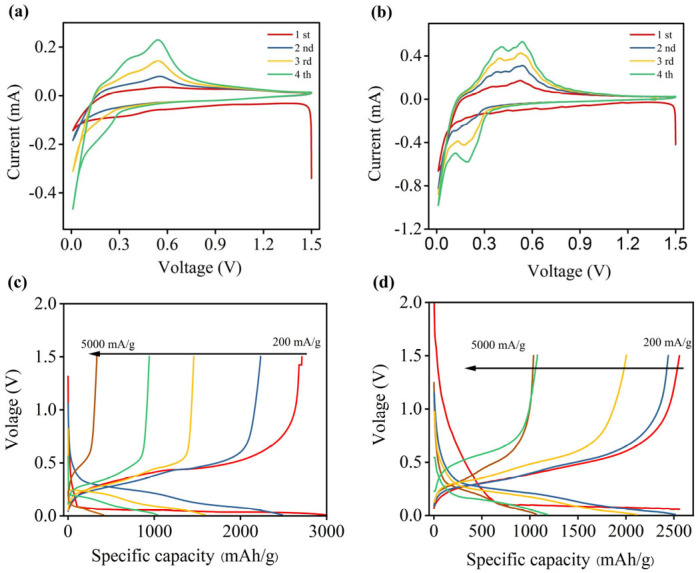
Cyclic voltammograms and charge–discharge curves of Si electrode with CMC binder (**a**,**c**) and CMC−SF6 binder (**b**,**d**).

Figure 4 and Figure S5 show the cyclic voltammograms of the CMC and CMC-SF-6 binders, respectively. In the first cycle, a broad reduction peak around 0.6 V is observed, corresponding to the formation of the SEI layer. This peak disappears in subsequent cycles. Additionally, a reduction peak around 0.1 V during the first discharge process is attributed to the alloying reaction between crystalline silicon and lithium, which is consistent with the charge–discharge curves. In subsequent cycles, the reduction peak shifts to around 0.2 V, likely due to the lithiation process of amorphous silicon. During the charging process, two oxidation peaks at 0.3 V and 0.5 V are observed, corresponding to the delithiation process of the silicon–lithium alloy at different stages. The positions of the redox peaks are consistent with the charge–discharge curves (Figure 4c,d). The first-cycle Coulombic efficiency increases from 90.8% for the CMC binder to 93.1% for the SF-CMC-6 composite binder. This improvement can be attributed to the superior mechanical properties and flexibility of the composite binder, as well as the abundant polar groups (-NH_2_, -COOH) in SF, which strengthen the interfacial adhesion between the electrode and current collector.

**Figure 5:**
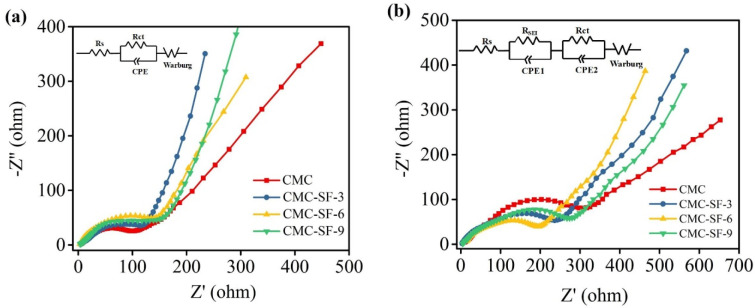
Impedance spectra before (**a**) and after (**b**) cycling.

EIS tests were conducted on half-cells before cycling and after 50 cycles (Figure 5). The equivalent circuit diagram is shown in the inset of Figure 5, with the corresponding values of solution resistance (R_s_), SEI layer resistance (R_SEI_), and charge-transfer resistance (R_ct_) listed in Table 2. As shown in Figure 5, the Nyquist plots before cycling exhibited a semicircle in the medium-frequency region, representing the total resistance (including ohmic resistance, contact resistance, and charge transfer resistance). Table 2 reveals that the R_ct_ of SF-modified CMC composite binders increased before cycling. This can be attributed to the lower percent mass change and reduced electrolyte absorption of the SF-modified CMC composite binders in the electrolyte. After 50 cycles, two distinct semicircles were observed in the Nyquist plots for all samples: one in the high-frequency region corresponding to R_SEI_, and another in the mid-frequency range representing R_ct_. Notably, the SF-CMC composite binders demonstrated significantly lower R_SEI_ values compared to the pure CMC binder. This observation highlights the superior mechanical properties of the SF-modified CMC binders—their cross-linked structure effectively suppresses the severe volume expansion of silicon nanoparticles, thereby mitigating resistance increases. Furthermore, these binders help maintain more complete electrical interactions between active components in the electrode, which plays a crucial role in delaying resistance growth.

**Figure 6:**
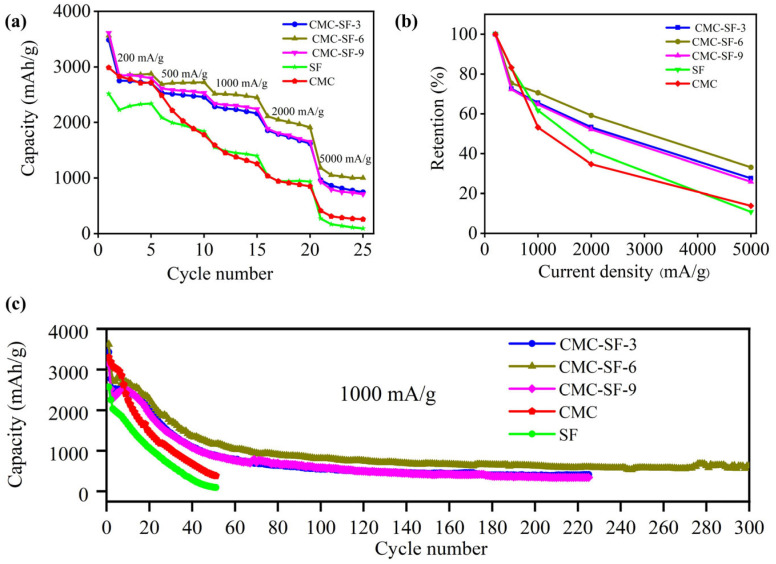
Electrochemical performance: rate performance (**a**); capacity retention rate (**b**); cycle performance (**c**).

The rate performance at different current densities is shown in Figure 6a. The results demonstrate that the capacity retention of CMC-SF samples is significantly improved compared to CMC and SF electrodes. The initial discharge capacities at 200 mA/g were 2987 mAh/g for CMC, 2516 mAh/g for SF, 3485 mAh/g for CMC-SF-3, 3564 mAh/g for CMC-SF-6, and 3612 mAh/g for CMC-SF-9. The initial Coulombic efficiency increased with the SF content. At higher current densities, CMC and SF electrodes exhibited significant capacity decay, with capacities of only 412 mAh/g and 271 mAh/g, respectively, at 5000 mA/g. In contrast, the SF-modified CMC-SF samples show slower capacity decay. As the SF content increased from 3% to 9%, the capacity retention initially improved and then decreased. At 5000 mA/g, the capacities of CMC-SF-3, CMC-SF-6, and CMC-SF-9 were 964 mAh/g, 1182 mAh/g, and 935 mAh/g, respectively. The results indicate that SF-modified CMC-SF improves the rate performance of silicon-based electrodes, with the 6% SF sample exhibiting the best performance across different current densities.

Figure 6c presents the cycling performance of various electrodes at a current density of 1000 mA/g, with the performance of SF as a binder shown under the same conditions for comparison. After 50 cycles, the capacity of the SF binder was only 102 mAh/g, indicating that SF alone cannot provide good electrochemical cycling performance. The CMC binder retained a reversible capacity of 400 mAh/g after 50 cycles, corresponding to a capacity retention rate of merely 12.1%. In contrast, the SF-modified CMC-SF binders demonstrated significantly improved cycling performance. Although the specific capacity declined rapidly during the initial cycles—attributed to SEI film formation and silicon particle pulverization, the cycling behavior stabilized as the SEI film matured. After 50 cycles, the CMC-SF-3, CMC-SF-6, and CMC-SF-9 electrodes delivered reversible capacities of 982 mAh/g, 1138 mAh/g, and 986 mAh/g, respectively. Moreover, the CMC-SF-6 sample maintained a capacity of 620 mAh/g after 300 cycles. These results demonstrate that the incorporation of SF significantly enhances the electrochemical cycling performance of the binder, with the 6% SF formulation exhibiting the best performance. However, when the SF content increased to 9%, the cycling performance of the binder deteriorated, suggesting that 6% SF is the optimal content. Overall, the CMC-SF binders offer superior adhesion, mechanical properties, and electrochemical performance compared to pure CMC. The 6% SF composition stands out as having the best overall performance, including enhanced rate capability and cycling stability, making it the optimal choice for silicon-based electrodes.

**Figure 7:**
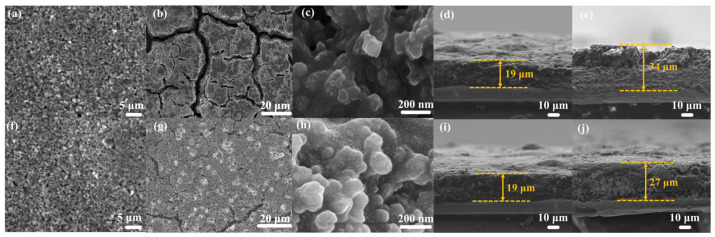
SEM images of electrode before and after cycling of CMC binders (**a**–**e**) and CMC-SF-6 binders (**f**–**j**).

To evaluate the ability of the binders to accommodate large volume changes during cycling, the cycled batteries were disassembled in a glove box, and the electrode sheets were extracted. The electrodes were washed in DMC solvent to remove residual electrolytes and then dried. The morphological changes in the electrodes before and after cycling were observed using SEM for both CMC and CMC-SF-6 binders. Figure 7a,b shows the top-view SEM images of the CMC electrode before (Figure 7a) and after (Figure 7b) cycling, including both macroscopic and locally magnified views. The initial CMC electrode surface showed no cracks, while after 50 cycles, the CMC electrode exhibited numerous wide and deep cracks. A closer examination of the local area (Figure 7c) revealed that the silicon particles were completely covered by a thick SEI layer, making it difficult to distinguish the morphology of the silicon particles. This indicates that the volume expansion of silicon during cycling lead to the repeated cracking and reformation of the SEI layer, resulting in the silicon particles being enveloped by a thick SEI film. The growth of this thick SEI layer also consumed a significant amount of electrolyte, leading to rapid capacity decay during cycling. Cross-sectional SEM images show that the electrode employing the CMC binder swelled from 19 µm (before cycling Figure 7d) to 34 µm (after cycling Figure 7e) and exhibited structural collapse. In contrast, for the CMC-SF-6 composite binder, the top-view SEM images of the electrode before (Figure 7f) and after (Figure 7g) cycling show that the electrode surface remained crack-free before cycling, and only a few cracks with reduced depth and width were observed after cycling compared to the CMC binder. A locally magnified view (Figure 7h) clearly reveals the morphology of the silicon particles, indicating that the prepared binder effectively mitigated the adverse effects of silicon volume expansion, maintaining a stable electrode structure after cycling. Cross-sectional SEM images show that the electrode employing CMC-SF-6 binders swelled from 19 µm (before cycling Figure 7i) to 34 µm (after cycling Figure 7j) and exhibited a relatively small change in electrode thickness. This result confirms that our SF-modified CMC binder effectively alleviates volume expansion, as it maintains structural integrity with a markedly lower swelling ratio compared to the unmodified binder.

**Figure 8:**
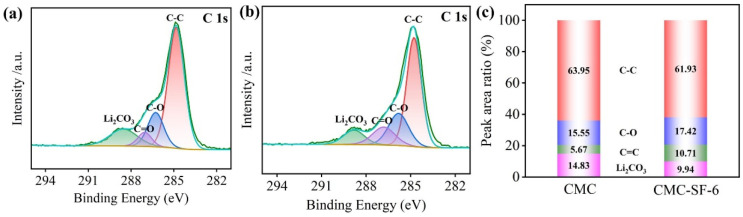
XPS spectra of C1s of CMC binder (**a**); CMC-SF-6 binder (**b**); C1s peak area ratio of CMC binder and CMC-SF-6 binder (**c**).

To investigate the working mechanism of the binder, we performed XPS analysis on the SEI film after battery cycling. As shown in the C1s spectrum of the SEI film after 100 cycles (Figure 8), the Li_2_CO_3_ content decreased from 14.83% with the CMC binder to 9.94% when using CMC-SF-6 as the binder. This indicates that the SEI formed with the CMC-SF-6 binder exhibited improved integrity [28,29].

**Figure 9:**
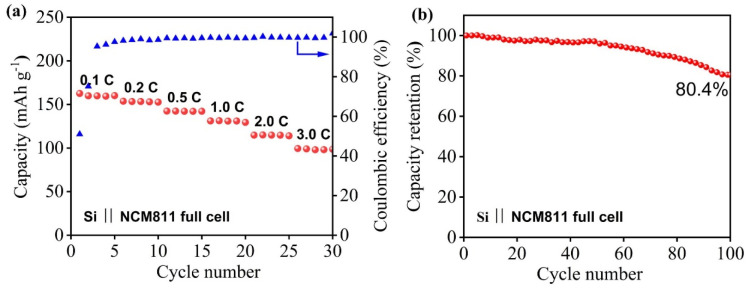
Rate capabilities (**a**) and cycling characteristics (**b**) of full cells with Si anode and NCM811 cathode.

To further evaluate the practical application potential of the CMC-SF composite binders, Si-based full cells paired with an NCM811 cathode were assembled and tested at a voltage range of 2.5–4.2 V. The Figure 9a rate capability test revealed that the full cell delivered specific capacities of 160, 153, 141, 131, 115, and 98 mAh/g at 0.1 C, 0.2 C, 0.5 C, 1.0 C, 2.0 C, and 3.0 C, respectively (with 1 C = 200 mA/g based on the cathode active material), demonstrating favorable rate performance. Furthermore, as shown in Figure 9b, the cell retained 80.4% of its capacity after 100 cycles at 1 C, indicating excellent cycling stability and promising potential for practical applications.

## 4. Conclusions

Through the molecular design of incorporating SF into the water-soluble CMC binder, we successfully developed a robust binder for high-performance silicon-based anodes. The optimal binder with 6% SF content delivers exceptional electrochemical performance, demonstrating a high specific capacity of 1182 mAh/g even at a high current density of 5000 mA/g and a good cycling stability with a capacity retention of 620 mAh/g after 300 cycles for the CMC-SF-6 sample at 1000 mA/g. The CMC–SF binder enables silicon-based full cells (vs. NCM811) to achieve high-rate capability (160 to 98 mAh/g from 0.1 C to 3.0 C) and excellent cycle stability (80.4% retention after 100 cycles). This enhancement is attributed to the significantly improved mechanical properties of the binder and its strengthened interaction with silicon particles, as designed. Therefore, this SF-modified CMC binder presents a highly promising strategy for enabling the practical application of silicon-based electrodes in next-generation lithium-ion batteries.

## Figures and Tables

**Table 1:** The percent mass change in different binders in electrolyte.

Sample	Mass Change (%)	Solubility
CMC-SF-9	5.13	Insoluble
CMC-SF-6	5.86	Insoluble
CMC-SF-3	6.93	Insoluble
CMC	8.12	Insoluble

**Table 2:** The corresponding values of R_s_, R_SEI_, and R_ct_ of electrode before and after cycling.

	Before Cycling		After Cycling		
	R_s_	R_ct_	R_s_	R_ct_	R_SEI_
CMC	3.812	134.8	8.078	233.0	352.4
CMC-SF-3	6.120	148.2	6.837	142.4	263.5
CMC-SF-6	4.667	175.1	8.414	139.5	241.2
CMC-SF-9	4.328	189.9	7.797	157.4	323.3

## Data Availability

Data will be made available on request.

## Supplementary Materials

The following supporting information can be downloaded at: https://www.mdpi.com/article/10.3390/nano15191509/s1, Figure S1. Preparation process flowchart and physical images, Figure S2. Peel test of electrode sheet with CMC and CMC-SF composite binder, Figure S3. Isothermal dynamic frequency sweep plot, Figure S4. Contact angle tests using different binders and a liquid electrolyte as the substrate and solvent, respectively, Figure S5. CV curves of different binder samples.
