# Supplementary material for: Preparation of Silk Fibroin–Carboxymethyl Cellulose Composite Binder and Its Application in Silicon-Based Anode for Lithium-Ion Batteries
Source: Nanomaterials (Basel). 2025 Oct 2;15(19):1509. doi: 10.3390/nano15191509 (PMC12525972; doi:10.3390/nano15191509)
Supplement: Supplementary file 1 [file nanomaterials-15-01509-s001.zip › nanomaterials-3865536-supplementary.pdf]

Preparation of silk fibroin-carboxymethyl cellulose composite binder and its application in silicon-based anode for lithium-ion battery

Shuai Huang [1,3], Ruyi Wang [2], Mingke Lei [1,3], Qingxuan Geng [4], Qingwei Li [4,*], Jiwei Zhang [2,*] and Jingwei Zhang [2]

[1] School of Environmental Engineering, Yellow River Conservancy Technical University, Kaifeng, 475004, China

[2] National & Local Joint Engineering Research Center for Applied Technology of Hybrid Nanomaterials, Henan University, Kaifeng 475004, China

[3] Henan Engineering Technology Research Center of Green Coating Materials, Kaifeng, 475004, China

[4] State Key Laboratory of Green Papermaking and Resource Recycling, Advanced Research Institute for Multi-disciplinary Science, Qilu University of Technology (Shandong Academy of Sciences), Daxue Road 3501, Ji-nan 250307, Shandong Province, China

* Correspondence: liqingwei@qlu.edu.cn; zhangjiwei@henu.edu.cn

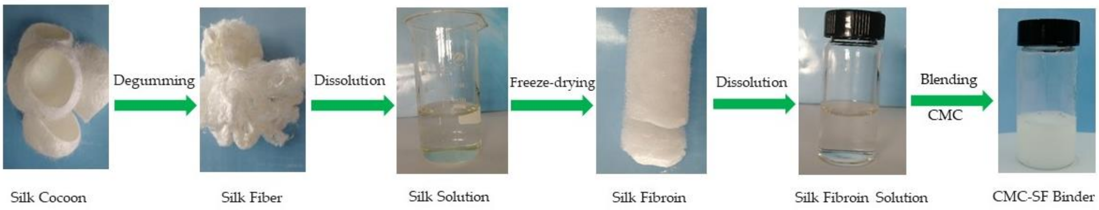

Figure S1. Preparation process flowchart and physical images

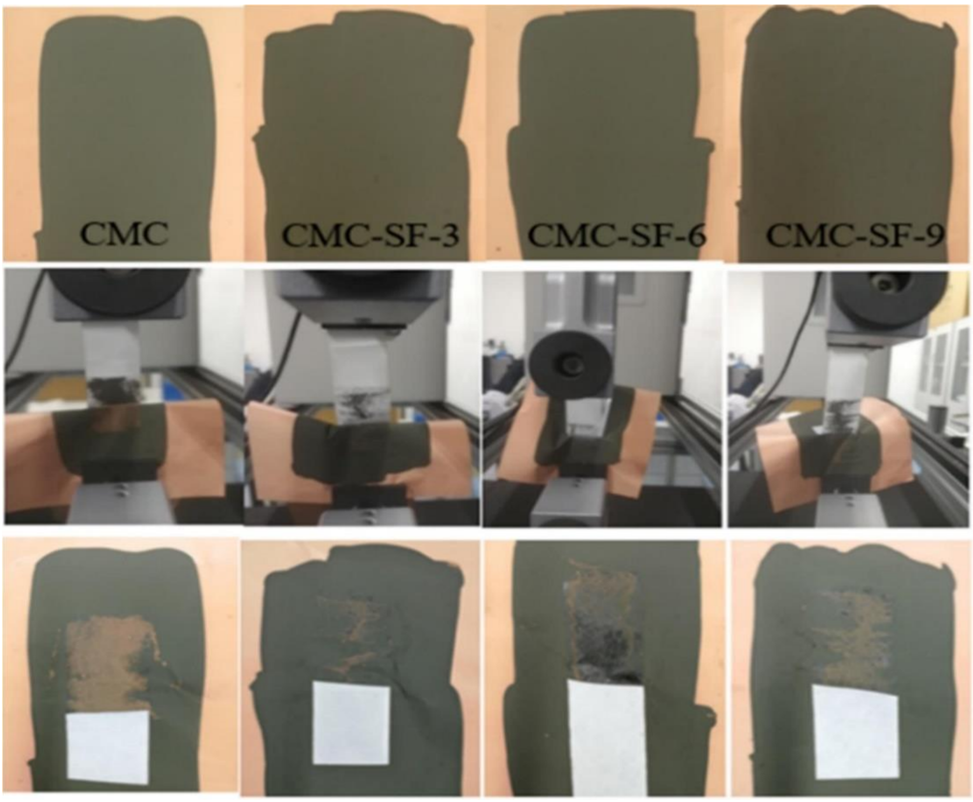

Figure S2. Peel test of electrode Sheet with CMC and CMC-SF composite binder

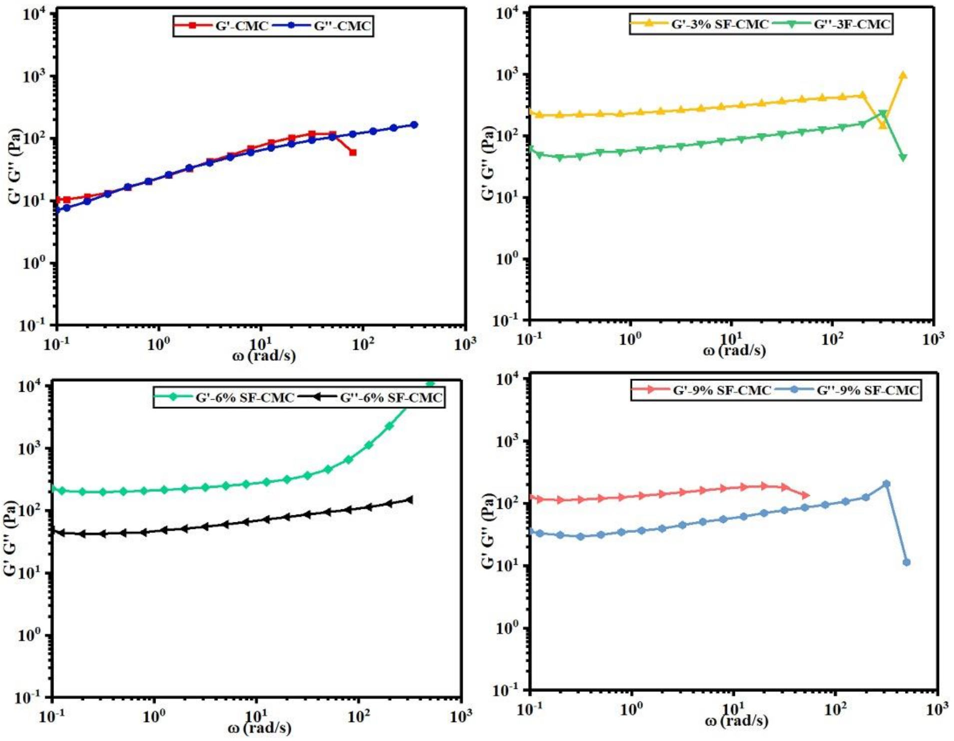

Figure S3. Isothermal dynamic frequency sweep plot

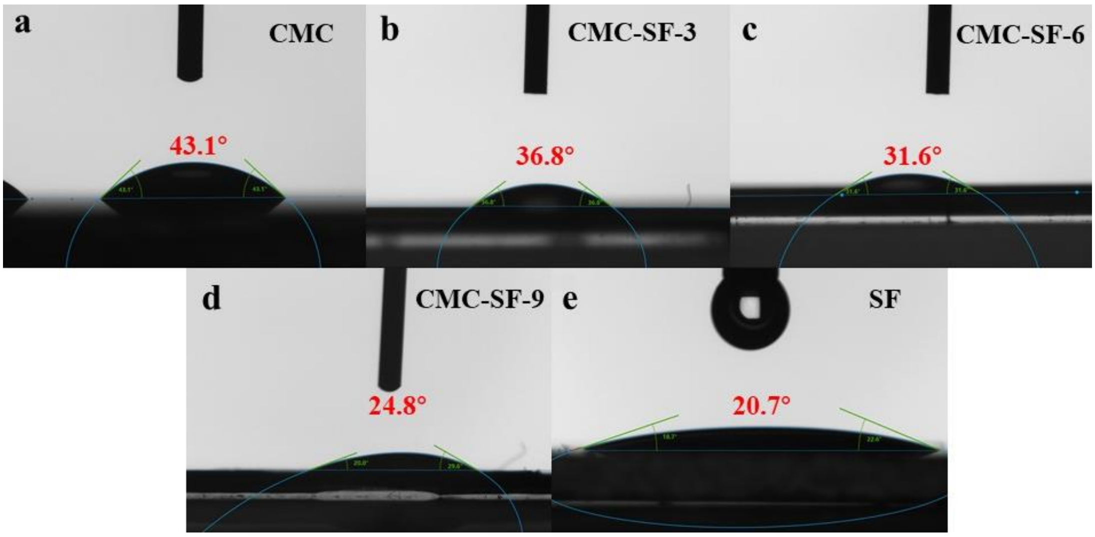

Figure S4. Contact angle tests using different binders and a liquid electrolyte as the substrate and solvent, respectively.

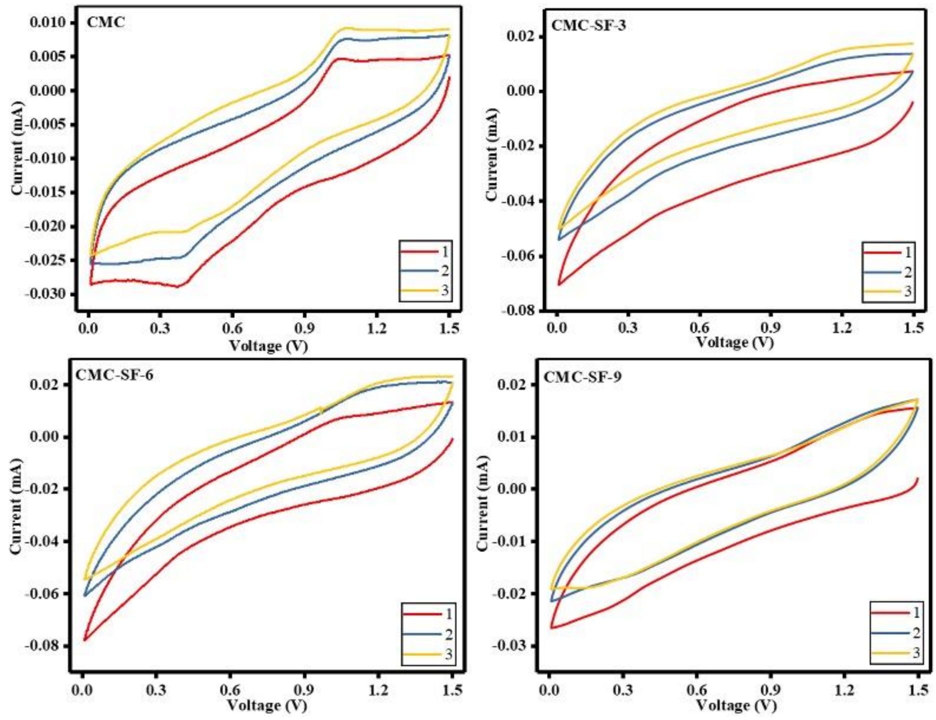

Figure S5. CV curves of different binder samples
